# Development of Self-Consistency Models of Anticancer Activity of Nanoparticles under Different Experimental Conditions Using Quasi-SMILES Approach

**DOI:** 10.3390/nano13121852

**Published:** 2023-06-13

**Authors:** Andrey A. Toropov, Alla P. Toropova, Danuta Leszczynska, Jerzy Leszczynski

**Affiliations:** 1 Laboratory of Environmental Chemistry and Toxicology, Istituto di Ricerche Farmacologiche Mario Negri IRCCS, Via Mario Negri 2, 20156 Milano, Italy; alla.toropova@marionegri.it; 2 Interdisciplinary Nanotoxicity Center, Department of Civil and Environmental Engineering, Jackson State University, 1325 Lynch Street, Jackson, MS 39217-0510, USA; danuta@icnanotox.org; 3 Interdisciplinary Nanotoxicity Center, Department of Chemistry, Physics and Atmospheric Sciences, Jackson, MS 39217-0510, USA; jerzy@icnanotox.org

**Keywords:** nanoparticle, anticancer activity, QSAR, quasi-SMILES, Monte Carlo method, CORAL software

## Abstract

Algorithms of the simulation of the anticancer activity of nanoparticles under different experimental conditions toward cell lines A549 (lung cancer), THP-1 (leukemia), MCF-7 (breast cancer), Caco2 (cervical cancer), and hepG2 (hepatoma) have been developed using the quasi-SMILES approach. This approach is suggested as an efficient tool for the quantitative structure–property–activity relationships (QSPRs/QSARs) analysis of the above nanoparticles. The studied model is built up using the so-called vector of ideality of correlation. The components of this vector include the index of ideality of correlation (*IIC*) and the correlation intensity index (*CII*). The epistemological component of this study is the development of methods of registration, storage, and effective use of experimental situations that are comfortable for the researcher-experimentalist in order to be able to control the physicochemical and biochemical consequences of using nanomaterials. The proposed approach differs from the traditional models based on QSPR/QSAR in the following respects: (i) not molecules but experimental situations available in a database are considered; in other words, an answer is offered to the question of how to change the plot of the experiment in order to achieve the desired values of the endpoint being studied; and (ii) the user has the ability to select a list of controlled conditions available in the database that can affect the endpoint and evaluate how significant the influence of the selected controlled experimental conditions is.

## 1. Introduction

Knowledge is the basis of all actions aimed at improving people’s lives and the evolution of civilization as a whole. However, knowledge has internal contradictions. For example, in order to manage or even observe complex processes, it is necessary first to study the available information, which is the personification of the corresponding knowledge. If the knowledge is not structured, learning to use these disordered facts or skills becomes quite expensive and difficult in several respects, such as the necessity of a long period to learn and apply expensive equipment and software. The main function of the quasi-SMILES conception examined here is to search for reasonably simple ways to study complex phenomena. The simulation of physicochemical and biochemical behavior nanomaterials is quite a complex phenomenon.

Strangeness is one of the manifestations of reality about which it is difficult to speak clearly. Nevertheless, strangeness is often a property of things that are new, unexpected, or important. For the implementation of any activity, an economy of thinking is necessary. In practice, such savings can be achieved in various ways. Sorting is perhaps the simplest fragment of the thought economy process. Sorting consists of selecting the most informative of the data under experimental conditions in the laboratory, production, and interaction with environmental circumstances (climate, epidemics, economic crises). However, sorting does not provide any guidance for decision making at the stage when the choice of priorities is made. Models are needed at this stage. Having a model for a process can help one to manage the process. The complexity of the choice is considerable because usefulness and harm can change places when harm becomes a benefit and the benefit turns out to be harmful. For instance, the toxicity of nanoparticles is considered a useful quality because it can be used for good aims. However, left unattended, this toxicity can harm or even kill humans and animals. The list of nanoparticles is expanding exponentially. The number of types of toxicity is by no means small. Obviously, under such circumstances, it is impossible quickly to evaluate experimentally all nanoparticles that are used or can be used. However, the assessment of the physicochemical and biochemical behavior of a significant number of new nanoparticles using databases on already studied nanoparticles is quite feasible. The key points in the development of such models are the need to reduce the memory and logic requirements of the users of the models. In other words, developers of models should provide user-friendly means of evaluating new nanoparticles. At the same time, it is highly desirable and important that such models consider the effect of possible changes in the corresponding directions for the experimental use of nanoparticles [1].

Quantitative structure–property–activity relationships (QSPRS/QSARs) are a well-known approach to establishing models of different endpoints considered as a mathematical function of molecular structure. A successful QSAR analysis is possible if and only if: (i) there is a large enough number of compounds with a clear definition of the congeneric features corresponding to the molecules; (ii) there is a hypothesis on how and which molecular features affect the endpoint (topological architecture, 3D configurations, quantum mechanics interactions, etc.); and (iii) checking of the predictive potential of the model can be carried out [2,3]. However, the QSPR/QSAR paradigm is widely applied to relatively traditional substances, such as organic, inorganic, metal-organic chemicals, and polymers. On the other hand, attempts to use the abovementioned paradigm for nanomaterials face quite a complex situation. First, there are only small databases on experimentally measured basic endpoints, such as thermodynamic parameters and/or biochemical effects. In other words, selecting a series of nanomaterials with experimental data is the problem. Secondly, the huge number of atoms in the majority of nanomaterials lessens the usefulness of traditional molecular descriptors: their values become non-sensitive to small molecular modifications.

There is an urgent need to clarify the approaches and methodology for measuring the biochemical potential of engineered nanomaterials. Factually, this is a problem of tuning computational and experimental approaches oriented to “traditional” substances for application to nanomaterials. The possibility of employing computational approaches like nano-QSAR or nano-read-across to predict nanomaterial hazards based on some “standard” databases is an attractive possibility from a financial point of view. The attractiveness from an ethical point of view is also clear (minimal animal tests). Many research studies have endeavored to investigate the eco-toxicological hazards of engineered nanomaterials. However, little is known regarding nanomaterials’ actual environmental risks, combining hazard and exposure data on a planetary scale [1].

It has been assumed that strangeness and research activity rarely intersect. However, when they meet, they either reinforce or disregard each other. For example, modelling, one of the most important and complex areas of research, can be summed up in the short aphorism “All models are wrong, but some are useful” [4].

**Figure 1:**
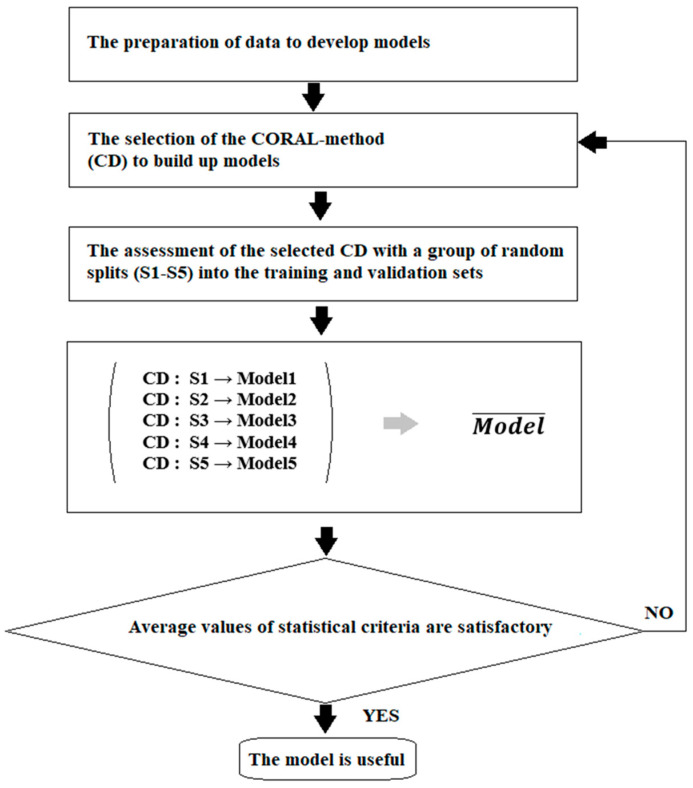
General scheme for building up the models examined here.

Systematization of knowledge related to nanomaterials has become necessary due to the fast growth of applications of these “unusual” substances. Systematization involves various aspects of research activity. The development of approaches that allow for the simulation of different characteristics of nanomaterials, including their interactions with other species, is one of them. There are many methods to perform such simulations. One of the possible approaches is to carry simulations out using the so-called quasi-SMILES [5,6,7,8,9,10,11,12,13] approach. The traditional simplified molecular input line entry system (SMILES) [14] allows the molecular architecture to be represented via a sequence of symbol-codes. At the same time, the quasi-SMILES approach gives us the possibility of representing the experimental conditions or even any arbitrary eclectic data related to the behavior of nanomaterials via symbol-codes. Figure 1 displays the general scheme for the simulation of the biological effects of nanoparticles. This scheme was used to build up the models described below.

## 2. Materials and Methods

### 2.1. Data

The dataset used in this study includes measurements of half maximal effective (EC50), inhibitory (IC50), and lethal (LC50) concentration toxicity endpoints toward cell lines A549 (lung cancer), THP-1 (leukemia), MCF-7 (breast cancer), Caco2 (cervical cancer), and hepG2 (hepatoma) under different experimental conditions (various nanoparticles, size, exposure time) for human cells. The indicated conditions and circumstances were represented by special codes listed in Table 1. These codes are used for the construction of the quasi-SMILES that represent the above measurements of the toxicity of the studied nanoparticles [15].

The listed codes for quasi-SMILES make it possible to constructively describe the available experimental situations for developing models in order to predict the results of varying codes (i.e., varying of an experiment). The system described can assess the statistical significance of individual experimental conditions (i.e., the above codes for quasi-SMILES). In other words, concentration values, exposure times, impacted objects, nanoparticle sizes, and others are under consideration to simulate the behavior of nanoparticles.

After removing duplicates, the source [15] contains 935 measurements, representing data related only to human cells. The total set studied here includes 102 measurements. These data were randomly split into an active training set (≈25%), a passive training set (≈25%), a calibration set (≈25%), and a validation set (≈25%). The advantages of considering a structured training set (divided into an active training set, passive training set, and calibration set) are described in the literature [16]. Five such splits that involve the deposition of different data each time for the considered data sets are considered to assess the reproducibility of the approach considered here for creating models [17].

### 2.2. Optimal Descriptor

The optimal descriptor is the sum of the correlation weights of the quasi-SMILES codes obtained by the Monte Carlo method using the CORAL software (http://www.insilico.eu/coral, accessed on 29 May 2023). The values of the optimal descriptor serve as the basis for the model of half-maximal concentration (*HMC*) (i.e., EC50, IC50, or LC50) calculated by the formula:

(1) $${HMC}_{k}=C_{0}+C_{1}\times DCW(T,N)$$

The optimal descriptor depends on the selected method of the Monte Carlo optimization of the correlation weights for codes of quasi-SMILES (Table 1). The *T* and *N* are the parameters of the optimization procedure. *T* is a threshold applied to define rare codes. If *T* = 1, this means that codes which are absent in the active training set are rare. The rare codes are not involved in modelling (their correlation weights are zero). *N* is the number of epochs of the Monte Carlo optimization.

### 2.3. Optimization of Correlation Weights

The optimal descriptors are calculated using the correlation weights obtained by the Monte Carlo optimization [16,17]. Two target functions of the optimization are compared here:

(2) $${TF}_{1}=r_{AT}+r_{PT}-\left|r_{AT}-r_{PT}\right|\times 0.1$$

(3) $${TF}_{2}=r_{AT}+r_{PT}-\left|r_{AT}-r_{PT}\right|\times 0.1+(IIC+CII)\times 0.3$$

*r_AT_* and *r_PT_* are correlation coefficients between the experimental and predicted values for the active and passive training sets, respectively. The *IIC* represents the index of ideality of correlation [15,16,17]. The *CII* is the correlation intensity index [15,16,17].

**Figure 2:**
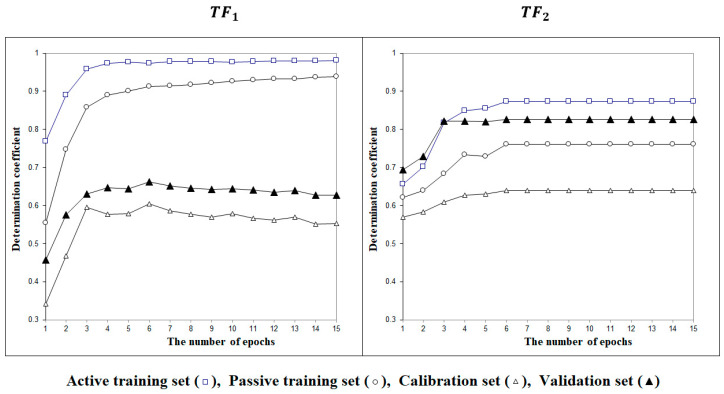
The history of the Monte Carlo optimization carried out using the target functions *TF*_1_ or *TF*_2_.

Figure 2 contains examples of the optimization history with target functions *TF*_1_ and *TF*_2_. The figure demonstrates the advantage of the target function *TF*_2_ graphically.

### 2.4. Mechanistic Interpretation

If Monte Carlo optimization is carried out several times, then some components of the optimized correlation weights will have positive values in all optimization trials. Such correlation weights indicate those fragments of quasi-SMILES that are growth promoters of the studied endpoint. At the same time, some of the correlation weights will only have negative values. These correlation weights indicate those fragments of quasi-SMILES that are patrons of the decrease in the simulated endpoint. Correlation weights with alternating values (positive and negative in different runs of Monte Carlo optimizations) have no mechanical interpretation for the models under consideration.

### 2.5. Applicability Domain

The applicability domain for the described model defines via the so-called statistical defects of codes used in quasi-SMILES. These defects can be calculated as follows:

(4) $$d_{k}=\frac{\left|{P(S}_{k})-{P\prime (S}_{k})\right|}{N\left(S_{k}\right)+N\prime (S_{k})}+\frac{\left|{P(S}_{k})-{P\prime \prime (S}_{k})\right|}{N\left(S_{k}\right)+N\prime \prime (S_{k})}+\frac{\left|{P\prime (S}_{k})-{P\prime \prime (S}_{k})\right|}{N\prime \left(S_{k}\right)+N\prime \prime (S_{k})}$$

where *P*(*S_k_*), *P*′(*S_k_*) *P*″(*S_k_*) are the probability of *S_k_* in the active training set, passive training set, and calibration set, respectively; *N*(*S_k_*), *N*′(*S_k_*), and *N*″(*S_k_*) are frequencies of *S_k_* in the active training set, passive training set, and calibration set, respectively. The statistical defects of quasi-SMILES (*D_j_*) are calculated as follows:

(5) $$D_{j}=\sum_{k=1}^{NA}d_{k}$$

where *NA* is the number of non-blocked codes in quasi-SMILES.

A quasi-SMILES falls in the applicability domain if

$$Dj<2\times \bar{D}$$

## 3. Results

Table 2 contains an example of the model of biological activity related to different experimental situations represented via quasi-SMILES (split 1, target function *TF*_2_). However, since the statistical characteristics of a model can vary for different splits into the training and validation set, it is necessary to consider a system of several different splits.

Two CORAL methods are applied here for five random splits.

***The first CORAL method*** is the Monte Carlo optimization with target function without the vector of ideality of correlation and the correlation weights of fragments of local symmetry (Equation (2)). This method gives the models represented in Table 3.

***The second CORAL method*** is the Monte Carlo optimization with target function calculated by Equation (3) with the use of the vector of ideality of correlation together with the correlation weights of fragments of local symmetry. This method gives the models represented in Table 4.

One can see that the statistical characteristics of models observed in the case when the second method is applied are better than those observed in the case of the first method. This is evidenced by the average determination coefficient for the validation set, which in the case of the first method amounts to $\overset{-}{R_{V}^{2}}=0.605(∆R_{V}^{2}=0.073$). The second method gave $\overset{-}{R_{V}^{2}}=0.751(∆R_{V}^{2}=0.097$).

## 4. Discussion

The most popular traditional QSAR modelling approach can be formulated as follows: (i) selection of a group of available and convenient descriptors; (ii) defining a model using training-set substances; and (iii) validating the model using external validation-set substances. One can formulate several questions related to the optimization of this approach. For example, how will the model’s statistical quality change in the next division into training and testing samples? How to avoid overfitting (i.e., how to avoid a situation where a good model for the training set becomes a bad model for external substances)? How can one estimate the probability of obtaining a satisfactory and reliable model? In fact, the approach under consideration attempts to solve these problems using original idealizations, assumptions, and limitations.

Much excellent research is dedicated to nano-topics; nevertheless, even a simple question, e.g., whether a nanomaterial can be assessed using software, is quite ambiguous. The results of different estimations can vary depending on the personal experience of the expert conducting the study, and one cannot guarantee the reproducibility of these assessments.

Perhaps the main and convenient (from the user point of view) idealization of the considered approach is that instead of searching for sources of numerous descriptors, it is supposed to use “artificial” optimal descriptors, which can be tuned to correlate with the endpoint of interest. This assumption may not be correct. In this case, the approach under consideration is unsuitable for such a task, and a useful alternative approach to solve the task becomes necessary. However, there are cases where the approach discussed here has been useful [6,7,8,9,10,11,12].

The approach considered here has various advantages. First, to apply this approach, one can use arbitrary data. There is no ‘a priori’ knowledge before the experiment about whether such data can improve the model or not. The instability of the values of the correlation weights is a reliable indicator of the uselessness of the tested factor. On the contrary, at the same time, stability is a significant indicator of the influence of the factor on the predictive potential of the model. Secondly, this approach makes it easy to change the set of correlation-weighted factors, thus radically changing the model. This facilitates fast evaluation of the benefits of various hypotheses related to the optimal list of factors involved in the model development process.

The universality of the approach provides the user with sample opportunities to choose a set of basic factors for developing a model. However, overextension of such a set can lead to useless models that are excellent for the training set of samples but are completely unsuitable for external sets of similar samples. Given this circumstance, it is difficult to formulate rules for dividing the available data into active learning, passive learning, calibration, and an external validation set. It seems reasonable to assume that each of the four mentioned sets has the same significance. Therefore, the distribution of available samples should be approximately the same, i.e., about 25% of the data for each set (Table 2 and Table 3).

The presented approach is similar to incremental methods based on the selection of suitable contributions from individual parts of molecules to describe or model the physicochemical property or biological activity of interest [18,19]. The main common feature of the described approach with the additive scheme is that in both cases, the modeled endpoint is considered as the sum of the contributions of some participants in the model-building process. The difference between the mentioned approaches lies in the fact that for the traditional additive scheme, the set of participants is constant. At the same time, for the quasi-SMILES approach, it is possible to vary the number and quality of participants in the model-building process. For example, theoretically, the user of the quasi-SMILES method can eliminate the correlation weights reflecting particle size by reducing the number of Monte Carlo optimization parameters.

On the other hand, the user can expand the brutto formulas by representing the corresponding metal oxides with traditional SMILES (e.g., instead of Al_2_O_3_ using [O-2].[O-2].[O-2].[Al+3].[Al+3]), thereby increasing the number of optimized parameters. Of course, such changes do not guarantee an improvement in the predictive potential of the model, but they do provide the user with extended opportunities in the search for a model of the phenomenon and perhaps even stimulate the user’s creative activity. Another important although hidden point is that the considered approach allows the user to identify and discard those quasi-SMILES fragments that are non-informative due to their low prevalence in training samples and/or in the general array of available data. This defines automatically through the appropriate selection of the threshold described above (i.e., parameter *T* in Equation (1)). Since QSAR is actually a random event [20] associated with and determined by the distribution of available data in training and control samples, this option is very useful because it allows one to go from so-called “naive cross-validation” to “two-step cross-validation” [21]. The difference between naive and two-step cross-validation is as follows. Naive cross-validation is the result of a single distribution in the training and the validation sets. In contrast, two-step cross-validation is the result of considering and analyzing multiple random distributions in the training and validation sets.

A very significant component of models built on optimal descriptors using quasi-SMILES codes is optimization procedures by the Monte Carlo method. The choice of the target function is the key to the success of such Monte Carlo calculations. The ideality index of correlations (*IIC*) [22] turned out to be a very useful finding for improving the objective functions for the Monte Carlo method optimizations used to construct optimal descriptors calculated in using both SMILES and quasi-SMILES codes. The majority of the phenomena involved in the natural sciences are complex. Idealization (or simplification) is one of the most common approaches to studying complex phenomena in the natural sciences, such as ideal gas, ideal solution, ideal crystals, and ideal symmetry [22]. Ideal correlation is also a very attractive variant of correlations in general. The main idea of ideal correlation expressed through *IIC* is a correlation with forced minimization of the mean absolute error (*MAE*). It is to be noted, however, that the application of *IIC* gives an improvement to the statistical characteristics for calibration and validation sets which is accompanied by reducing the correlation coefficient for the training sets. This is a paradox situation. Nevertheless, from a practical point of view, this situation is preferable to overtraining (i.e., the situation in which the excellent statistical quality for the training set is accompanied by poor statistical quality for the validation set). An analysis of the graphical representations of such a paradox observed with various geometric configurations on plots for ‘experiment vs calculation’ shows that such idealization is not always possible; fortunately, however, it is possible in the majority of cases of the different arrangement of points on the plot diagram ‘experiment vs calculation’ [23].

Another useful invention for improving the predictive potential of models based on quasi-SMILES codes is the Correlation Intensity Index (*CII*) [17]. Data on a group of quasi-SMILES (e.g., calibration set or validation set) with experimental and predicted values of an endpoint gives the possibility to estimate the contribution of each quasi-SMILES to the correlation between experiments vs calculated endpoint value. The negative effect of removing quasi-SMILES means it is a ‘supporter’ of the correlation; the positive effect of removing quasi-SMILES means it is an ‘oppositionist’ of the correlation. The sum of these effects is the *CII*.

## 5. Conclusions

The present study demonstrated that the quasi-SMILES technique gives statistically robust models for the half-maximal concentrations for the five cell lines. We showed that the statistical quality is well reproduced for five random splits of available data into a structured training set (i.e., the active training, passive training, and calibration sets) and an external validation set. Such approach is tested and recommended for various applications of the quasi-SMILES approach. Paradoxically, the vector of ideality of correlation, which is the sum of the described *IIC* and *CII*, improves the predictive potential of the studied models but in detriment to the statistical quality of the models on the training set. The described approach can be easily adapted to simulate other experimental situations and endpoints for nanomaterials and other substances (mixtures, polymers, peptides, proteins).

A quasi-SMILES approach describing experimental situations can be modified both by feedback (i.e., depending on the results obtained) and purely heuristically in accordance with spontaneous ideas for which statistical expertise is possible. Thus, quasi-SMILES are a simple and versatile approach for modelling experimental situations not yet implemented in practice. Indices *IIC* and *CII* cannot only improve Monte Carlo optimization, but the mentioned values can also be indicators of the predictive potential of various models.

## Figures and Tables

**Table 1:** Codes that are applied to construct quasi-SMILES.

Code	Comments	CW(Code)	d
[108m].......	Exposure time (minutes or hours)	−0.4525	0.0343
[12h].......	-	−0.3037	0.0513
[216].......	-	−0.1135	0.0251
[18h].......	-	0.0	1.0000
[24h].......	-	−0.2873	0.0063
[36h].......	-	0.0	1.0000
[48h].......	-	−0.3258	0.0160
[60h].......	-	0.0	1.0000
[72m]........	-	0.0	1.0000
[Ag]........	Types of nanoparticles	−0.4207	0.0178
[Al_2_O_3_].....	-	0.0	1.0000
[Bi_2_O_3_].....	-	0.0	1.0000
[CeO_2_]......	-	0.0	1.0000
[Co_3_O_4_].....	-	−0.3980	1.0000
[Co]........	-	0.0	1.0000
[Cu_2_O]......	-	0.0	1.0000
[CuO].......	-	0.4879	0.0333
[Cu]........	-	0.0	1.0000
[Fe_2_O_3_].....	-	0.0	1.0000
[MgO].......	-	0.0	1.0000
[Mn_3_O_4_].....	-	0.0	1.0000
[Mn_3_O_4_].....	-	0.0299	1.0000
[MoO_3_]......	-	0.0	1.0000
[NiO].......	-	0.0	1.0000
[Ni]........	-	0.0	1.0000
[Sb_2_O_3_].....	-	0.3814	0.0185
[SnO_2_]......	-	0.0	1.0000
[Y_2_O_3_]......	-	0.0	1.0000
[TiO_2_]......	-	0.4736	1.0000
[WO_3_].......	-	0.0	1.0000
[ZnO].......	-	0.0698	0.0338
[ZrO_2_]......	-	0.0	1.0000
[nm100].....	Size of nanoparticle in nm	0.0	1.0000
[nm11,9]....	-	0.0	1.0000
[nm12,2]....	-	0.0	1.0000
[nm14,9]....	-	0.0	1.0000
[nm−].......	-	−0.3009	0.0069
[nm149].....	-	0.0	1.0000
[nm14]......	-	0.0	1.0000
[nm19,7]....	-	0.0	1.0000
[nm20,8]....	-	0.0	1.0000
[nm20–30]...	-	0.0	1.0000
[nm22,9]....	-	0.0	1.0000
[nm20]......	-	0.4612	0.0160
[nm21]......	-	0.0	1.0000
[nm30–50]...	-	0.0	1.0000
[nm312].....	-	0.0	1.0000
[nm33,4]....	-	0.0	1.0000
[nm30]......	-	0.0	1.0000
[nm31]......	-	0.0	1.0000
[nm32]......	-	0.0	1.0000
[nm33]......	-	0.0	1.0000
[nm40–68]...	-	0.0	1.0000
[nm45,4]....	-	0.0	1.0000
[nm42]......	-	0.0	1.0000
[nm5–15]...	-	0.0	1.0000
[nm48,9]....	-	0.0	1.0000
[nm50]......	-	0.0	1.0000
[nm64–69]...	-	0.0	1.0000
[nm83–94]...	-	0.0	1.0000
[nm9,2].....	-	0.0	1.0000
[nm90]......	-	0.0	1.0000
[nm5].......	-	0.0	1.0000
[MCF-7].....	Cell line MCF-7 (breast cancer)	0.1888	1.0000
[A549]......	Cell line A549 (lung cancer)	0.1817	0.0068
[THP-1].....	Cell line THP-1 (leukemia)	0.3930	0.0185
[HepG2].....	Cell line hepG2 (hepatoma)	0.0	1.0000
[Caco2].....	Cell line Caco2 (cervical cancer)	−0.3912	0.0288
[EC50]......	concentration that gives 50% of the maximal response	0.3444	0.0164
[IC50]......	Concentration that gives 50% inhibition of a biological process	0.3023	0.0128
[LC50]......	Concentration that kills 50% test animals	0.0	1.0000
[LD50]......	Dose that kills 50% test animals	−0.3424	0.0010

**Table 2:** Quasi-SMILES, optimal descriptor *DCW(1,15)*, experimental and calculated biological activity, statistical defects (D) of quasi-SMILES, and applicability domain (AD).

*Set*	*ID*	*Quasi-SMILES*	*DCW(1,15)*	*Expr*	*Calc*	*D*	*AD*
P	5	[Ag][nm312][24h][IC50][THP-1]	2.2559	−3.5930	−3.6191	1.0553	YES
A	6	[Ag][nm5][24h][EC50][MCF-7]	−1.5770	−5.3340	−5.3221	2.0405	YES
C	7	[Ag][nm5][24h][EC50][HepG2]	−0.8541	−5.2630	−5.0009	2.0405	YES
P	8	[Ag][nm5][24h][EC50][A549]	−0.2091	−5.0330	−4.7143	1.0472	YES
C	9	[Ag][nm20][24h][EC50][A549]	2.7579	−4.0350	−3.3961	0.0632	YES
V	10	[Ag][nm50][24h][EC50][A549]	3.5230	−3.9100	−3.0562	1.0472	YES
A	11	[Ag][nm20][24h][EC50][MCF-7]	1.3899	−3.8780	−4.0039	1.0565	YES
P	12	[Ag][nm20][24h][EC50][HepG2]	2.1129	−3.6270	−3.6827	1.0565	YES
A	13	[Ag][nm50][24h][EC50][HepG2]	2.8780	−3.5070	−3.3427	2.0405	YES
P	14	[Ag][nm50][24h][EC50][MCF-7]	2.1550	−3.3560	−3.6640	2.0405	YES
P	15	[Al_2_O_3_][nm31][24h][ec50][A549]	5.0528	−2.0090	−2.3765	2.0171	YES
A	18	[Al_2_O_3_][nm40–68][24h][IC50][THP-1]	5.1653	−2.1810	−2.3265	2.0375	YES
A	21	[Bi_2_O_3_][nm149][24h][LC50][A549]	1.8978	−3.7930	−3.7782	3.0131	No
V	22	[Bi_2_O_3_][nm149][24h][LC50][HepG2]	1.2528	−3.6680	−4.0648	4.0063	No
C	23	[CeO_2_][nm14][24h][ec50][A549]	3.5063	−2.2360	−3.0636	2.0171	YES
P	25	[CeO_2_][nm33,4][24h][IC50][THP-1]	3.2467	−2.5570	−3.1789	2.0375	YES
P	26	[CeO_2_][nm-][24h][LD50][A549]	4.8429	−2.2360	−2.4697	1.0209	YES
C	27	[CeO_2_][nm-][48h][LD50][A549]	3.7565	−2.2360	−2.9524	1.0307	YES
P	30	[Co][nm20][24h][IC50][THP-1]	3.8171	−2.5540	−2.9255	1.0535	YES
A	31	[Co_3_O_4_][nm9,2][24h][IC50][Caco2]	3.3840	−3.2920	−3.1179	2.0478	YES
V	32	[Co_3_O_4_][nm9,2][24h][IC50][A549]	4.2995	−3.2650	−2.7112	2.0258	YES
A	33	[Co_3_O_4_][nm-][12h][ec50][A549]	3.0885	−3.3590	−3.2492	1.0689	YES
V	34	[Co_3_O_4_][nm-][108][ec50][A549]	2.9873	−3.3510	−3.2942	1.0519	YES
P	35	[Co_3_O_4_][nm-][36h][ec50][A549]	3.0404	−3.3480	−3.2706	2.0176	YES
V	36	[Co_3_O_4_][nm-][60h][ec50][A549]	3.1083	−3.3410	−3.2404	2.0176	YES
C	42	[Cu][nm90][24h][IC50][THP-1]	2.8851	−4.0500	−3.3396	2.0375	YES
C	44	[Cu][nm22,9][24h][IC50][THP-1]	3.0525	−2.7470	−3.2652	2.0375	YES
A	48	[Cu_2_O][nm83–94][24h][IC50][THP-1]	2.0570	−4.2440	−3.7075	2.0375	YES
V	49	[CuO][nm48][24h][ec50][A549]	3.4861	−2.9010	−3.0726	0.0504	YES
P	50	[CuO][nm11,9][24h][IC50][Caco2]	2.3263	−3.8000	−3.5879	1.0812	YES
C	51	[CuO][nm11,9][24h][IC50][A549]	3.2418	−3.6240	−3.1811	1.0592	YES
A	52	[CuO][nm42][18h][EC50][A549]	0.4290	−3.6000	−4.4308	2.0565	YES
C	58	[CuO][nm45,4][24h][IC50][THP-1]	3.0629	−3.8080	−3.2606	1.0709	YES
V	59	[CuO][nm30][24h][IC50][THP-1]	2.9429	−3.6480	−3.3139	1.0709	YES
V	64	[CuO][nm > 50][24h][IC50][A549]	3.1342	−3.4230	−3.2289	0.0592	YES
C	65	[CuO][nm-][60h][ec50][Caco2]	2.3452	−3.4190	−3.5795	1.0730	YES
C	66	[CuO][nm-][108][ec50][Caco2]	2.2242	−3.4020	−3.6332	0.1073	YES
V	67	[CuO][nm-][36h][ec50][A549]	3.1928	−3.3940	−3.2029	1.0510	YES
A	68	[CuO][nm-][108][ec50][A549]	3.1397	−3.3420	−3.2265	0.0852	YES
V	69	[CuO][nm-][216][ec50][Caco2]	1.8852	−3.3320	−3.7838	0.0981	YES
A	70	[CuO][nm-][216][ec50][A549]	2.8007	−3.3300	−3.3771	0.0761	YES
A	71	[CuO][nm-][60h][ec50][A549]	3.2607	−3.3280	−3.1727	1.0510	YES
C	72	[CuO][nm-][12h][ec50][A549]	3.2409	−3.3190	−3.1815	0.1022	YES
V	73	[CuO][nm-][36h][ec50][Caco2]	2.2773	−3.3140	−3.6096	1.0730	YES
A	74	[CuO][nm-][24h][ec50][A549]	4.0369	−3.2590	−2.8278	0.0573	YES
C	75	[CuO][nm-][24h][ec50][Caco2]	3.1214	−2.8320	−3.2346	0.0793	YES
V	76	[Fe_2_O_3_][nm39][24h][ec50][A549]	4.6853	−2.2040	−2.5397	1.0171	YES
V	77	[Fe_2_O_3_][nm-][24h][LD50][A549]	6.0495	−2.2040	−1.9337	1.0209	YES
A	78	[Fe_2_O_3_][nm-][48h][LD50][A549]	4.9631	−2.2040	−2.4164	1.0307	YES
A	79	[MgO][nm20][24h][ec50][A549]	6.4904	−1.6020	−1.7378	1.0331	YES
V	80	[Mn_3_O_4_][nm14,9][24h][IC50][Caco2]	2.6535	−3.5360	−3.4425	2.0478	YES
C	81	[Mn_3_O_4_][nm14,9][24h][IC50][A549]	3.5690	−3.2260	−3.0357	2.0258	YES
A	82	[Mn_3_O_4_][nm-][108][ec50][Caco2]	1.3911	−4.0440	−4.0034	1.0739	YES
V	83	[Mn_3_O_4_][nm-][216][ec50][Caco2]	1.0522	−3.8990	−4.1539	1.0648	YES
P	84	[Mn_3_O_4_][nm-][108][ec50][A549]	2.3066	−3.8570	−3.5966	1.0519	YES
V	85	[Mn_3_O_4_][nm-][60h][ec50][Caco2]	1.5121	−3.8390	−3.9496	2.0396	YES
P	86	[Mn_3_O_4_][nm-][60h][ec50][A549]	2.4276	−3.7670	−3.5428	2.0176	YES
A	87	[Mn_3_O_4_][nm-][216][ec50][A549]	1.9677	−3.6870	−3.7472	1.0428	YES
V	88	[Mn_3_O_4_][nm-][36h][ec50][A549]	2.3598	−3.4030	−3.5730	2.0176	YES
A	89	[MoO_3_][nm100][24h][ec50][A549]	5.4219	−2.1580	−2.2125	2.0171	YES
A	91	[Ni][nm64–69][24h][IC50][THP-1]	4.5318	−2.6220	−2.6080	2.0375	YES
P	94	[NiO][nm48,9][24h][IC50][THP-1]	2.7830	−4.5020	−3.3850	2.0375	YES
V	95	[Sb_2_O_3_][nm20,8][24h][IC50][Caco2]	1.7044	−3.7080	−3.8642	1.0663	YES
C	96	[Sb_2_O_3_][nm20,8][24h][IC50][A549]	2.6199	−3.5630	−3.4574	1.0443	YES
P	97	[Sb_2_O_3_][nm-][24h][ec50][Caco2]	2.2649	−4.4650	−3.6151	0.0644	YES
P	98	[Sb_2_O_3_][nm-][48h][ec50][Caco2]	1.1785	−4.4650	−4.0978	0.0741	YES
A	99	[Sb_2_O_3_][nm-][72][ec50][Caco2]	0.3668	−4.4650	−4.4585	1.0581	YES
A	100	[Sb_2_O_3_][nm-][216][ec50][A549]	1.9442	−4.1230	−3.7576	0.0612	YES
P	101	[Sb_2_O_3_][nm-][60h][ec50][Caco2]	1.4887	−3.8860	−3.9600	1.0581	YES
V	102	[Sb_2_O_3_][nm-][108][ec50][A549]	2.2832	−3.8800	−3.6070	0.0704	YES
C	103	[Sb_2_O_3_][nm-][108][ec50][Caco2]	1.3677	−3.7900	−4.0138	0.0924	YES
C	104	[Sb_2_O_3_][nm-][216][ec50][Caco2]	1.0287	−3.7820	−4.1644	0.0832	YES
V	105	[Sb_2_O_3_][nm-][60h][ec50][A549]	2.4042	−3.6940	−3.5533	1.0361	YES
P	106	[Sb_2_O_3_][nm-][36h][ec50][Caco2]	1.4208	−3.6410	−3.9901	1.0581	YES
V	107	[Sb_2_O_3_][nm-][36h][ec50][A549]	2.3363	−3.5380	−3.5834	1.0361	YES
P	108	[SnO_2_][nm21][24h][ec50][A549]	4.4986	−2.1790	−2.6227	2.0171	YES
P	111	[SnO_2_][nm33][24h][IC50][THP-1]	2.7228	−2.4290	−3.4117	2.0375	YES
A	112	[TiO_2_][nm30–50][24h][ec50][A549]	5.4703	−1.9030	−2.1910	2.0171	YES
P	114	[TiO_2_][nm5–15][24h][ec50][A549]	5.2689	−1.9030	−2.2805	2.0171	YES
P	120	[TiO_2_][nm12,2][24h][IC50][THP-1]	4.7545	−1.8770	−2.5090	2.0375	YES
V	121	[TiO_2_][nm-][24h][LD50][A549]	6.5859	−1.9030	−1.6953	1.0209	YES
A	122	[TiO_2_][nm-][48h][LD50][A549]	5.4994	−1.9030	−2.1780	1.0307	YES
C	123	[WO_3_][nm30][24h][ec50][A549]	3.4752	−2.3650	−3.0774	2.0171	YES
P	124	[Y_2_O_3_][nm33][24h][ec50][A549]	3.8498	−2.3540	−2.9110	2.0171	YES
A	126	[ZnO][nm21][24h][ec50][A549]	4.6905	−2.9080	−2.5375	1.0509	YES
P	127	[ZnO][nm19,7][24h][IC50][A549]	3.1916	−3.5120	−3.2034	1.0597	YES
C	128	[ZnO][nm19,7][24h][IC50][Caco2]	2.2761	−3.4280	−3.6102	1.0817	YES
V	132	[ZnO][nm53,6][24h][IC50][THP-1]	2.6149	−2.9990	−3.4596	0.0714	YES
P	133	[ZnO][nm-][48h][LD50][A549]	3.5956	−3.0330	−3.0239	0.0645	YES
C	134	[ZnO][nm-][24h][LD50][A549]	4.6821	−2.5110	−2.5412	0.0548	YES
V	139	[ZnO][nm > 50][24h][IC50][A549]	2.9661	−3.1300	−3.3036	0.0597	YES
P	140	[ZnO][nm-][216][ec50][A549]	2.6326	−3.2950	−3.4518	0.0766	YES
C	141	[ZnO][nm-][36h][ec50][A549]	3.0247	−3.2770	−3.2776	1.0515	YES
V	142	[ZnO][nm-][60h][ec50][A549]	3.0925	−3.2460	−3.2474	1.0515	YES
C	143	[ZnO][nm-][108][ec50][A549]	2.9715	−3.2380	−3.3012	0.0858	YES
V	144	[ZnO][nm-][60h][ec50][Caco2]	2.1770	−3.1890	−3.6542	1.0735	YES
C	145	[ZnO][nm-][216][ec50][Caco2]	1.7171	−3.1220	−3.8585	0.0986	YES
C	146	[ZnO][nm-][108][ec50][Caco2]	2.0560	−3.1130	−3.7079	0.1078	YES
C	147	[ZnO][nm-][36h][ec50][Caco2]	2.1092	−3.0900	−3.6843	1.0735	YES
A	148	[ZnO][nm-][12h][ec50][A549]	3.0727	−2.8380	−3.2562	0.1028	YES
C	149	[ZrO_2_][nm20–30][24 h][ec50][A549]	5.5291	−2.0900	−2.1649	2.0171	YES
A	150	[ZrO_2_][nm32][24 h][IC50][THP-1]	5.3352	−2.3340	−2.2510	2.0375	YES

**Table 3:** The statistical characteristics of models observed in the case of the first CORAL method.

*Split*	Set *	*n*	*R* ^2^	*CCC*	*IIC*	*CII*	*Q^2^*	*RMSE*	*F*
1	A	26	0.9953	0.9976	0.7316	0.9959	0.9946	0.062	5075
	P	25	0.8116	0.8577	0.7559	0.8444	0.7912	0.436	99
	C	25	0.5839	0.7129	0.5545	0.7389	0.5034	0.518	32
	V	26	0.6669	-	-	-	-	0.421	-
2	A	25	0.9674	0.9834	0.9079	0.9751	0.9625	0.162	682
	P	26	0.8741	0.8936	0.9141	0.9062	0.8592	0.452	167
	C	26	0.7119	0.8223	0.5094	0.7885	0.6510	0.287	59
	V	25	0.6664	-	-	-	-	0.440	-
3	A	26	0.9942	0.9971	0.9971	0.9947	0.9935	0.071	4145
	P	25	0.8097	0.8489	0.6615	0.8729	0.7840	0.488	98
	C	25	0.0250	0.1500	0.1577	0.8450	0.2846	0.654	1
	V	26	0.4691	-	-	-	-	0.459	-
4	A	26	0.9676	0.9836	0.7214	0.9690	0.9637	0.175	718
	P	25	0.9292	0.8513	0.2425	0.9471	0.9194	0.455	302
	C	25	0.4948	0.6813	0.5881	0.7106	0.3317	0.340	23
	V	26	0.6266	-	-	-	-	0.544	-
5	A	25	0.9958	0.9979	0.6653	0.9971	0.9948	0.061	5496
	P	25	0.8422	0.9095	0.8821	0.8796	0.8229	0.423	123
	C	26	0.5414	0.7188	0.4751	0.7746	0.4348	0.320	28
	V	26	0.5943	-	-	-	-	0.466	-

* A = active training set; P = passive training set; C = calibration set; V = validation set; *n* = the number of samples in a set; *R*^2^ = determination coefficient; *CCC* = concordance correlation coefficient; *IIC* = index of ideality of correlation; *CII* = correlation intensity index; *Q^2^* = leave-one-out cross-validated *R*^2^; *RMSE* = root mean squared error; *F* = Fischer F-ratio.

**Table 4:** The statistical characteristics of models observed in the case of the second CORAL method.

*Split*	Set *	*n*	*R* ^2^	*CCC*	*IIC*	*CII*	*Q^2^*	*RMSE*	*F*
1	A	26	0.9029	0.9490	0.6968	0.9203	0.8850	0.282	223
	P	25	0.7780	0.8048	0.8727	0.8391	0.7534	0.477	81
	C	25	0.5926	0.7273	0.7696	0.7677	0.5227	0.468	33
	V	26	0.6895	-	-	-	-	0.325	-
2	A	25	0.7646	0.8666	0.8072	0.8717	0.7154	0.434	75
	P	26	0.7538	0.7461	0.8669	0.8612	0.7185	0.622	73
	C	26	0.8353	0.8981	0.9124	0.8679	0.7857	0.221	122
	V	25	0.9132	-	-	-	-	0.239	-
3	A	26	0.8588	0.9240	0.5792	0.9002	0.8386	0.353	146
	P	25	0.7794	0.6971	0.5627	0.8848	0.7481	0.661	81
	C	25	0.6332	0.7749	0.7947	0.7882	0.5415	0.286	40
	V	26	0.6278	-	-	-	-	0.318	-
4	A	26	0.8868	0.9400	0.6906	0.9275	0.8671	0.327	188
	P	25	0.8327	0.8529	0.3663	0.8719	0.8021	0.486	114
	C	25	0.3795	0.5803	0.6157	0.8039	0.2497	0.439	14
	V	26	0.7355	-	-	-	-	0.413	-
5	A	25	0.8341	0.9095	0.8430	0.9063	0.8062	0.387	116
	P	25	0.7593	0.8271	0.4998	0.8316	0.7258	0.557	73
	C	26	0.8146	0.8062	0.9016	0.8982	0.7673	0.317	105
	V	26	0.7878	-	-	-	-	0.273	-

* A = active training set; P = passive training set; C = calibration set; V = validation set; *n* = the number of samples in a set; *R*^2^ = determination coefficient; *CCC* = concordance correlation coefficient; *IIC* = index of ideality of correlation; *CII* = correlation intensity index; *Q^2^* = leave-one-out cross-validated *R*^2^; *RMSE* = root mean squared error; *F* = Fischer F-ratio.

## Data Availability

Data are available within the article or in the Supplementary Materials (Tables S1 and S2).

## Supplementary Materials

The following supporting information can be downloaded at: https://www.mdpi.com/article/10.3390/nano13121852/s1. Supplementary Materials section contains technical details (Table S1 quasi-SMILES split 1–5 with experimental and calculated values of the half-maximal concentrations for the five cells lines; Table S2 includes the numerical data on the corresponding correlation weights for elements of quasi-SMILES).
